# Data analysis algorithm for the development of extracellular miRNA-based diagnostic systems for prostate cancer

**DOI:** 10.1371/journal.pone.0215003

**Published:** 2019-04-10

**Authors:** O. E. Bryzgunova, I. A. Zaporozhchenko, E. A. Lekchnov, E. V. Amelina, M. Yu. Konoshenko, S. V. Yarmoschuk, O. A. Pashkovskaya, A. A. Zheravin, S. V. Pak, E. Yu. Rykova, P. P. Laktionov

**Affiliations:** 1 The Laboratory of Molecular Medicine, Institute of Chemical Biology and Fundamental Medicine SB RAS, Novosibirsk, Russia; 2 The Laboratory of Biomedical Technologies, Centre of New Medical Technologies,“E. Meshalkin National Medical Research Center” of the Ministry of Health of the Russian Federation, Novosibirsk, Russia; 3 Federal State Autonomous Educational Institution of Higher Education "Novosibirsk National Research University", Novosibirsk, Russia; 4 Novosibirsk State Technical University, Novosibirsk, Russia; University of Minnesota Twin Cities, UNITED STATES

## Abstract

Urine of prostate cancer (PCa) carries miRNAs originated from prostate cancer cells as a part of both nucleoprotein complexes and cell-secreted extracellular vesicles. The analysis of such miRNA-markers in urine can be a convenient option for PCa screening. The aims of this study were to reveal miRNA–markers of PCa in urine and design a robust and precise diagnostic test, based on miRNA expression analysis. The expression analysis of the 84 miRNAs in paired urine extracellular vesicles (EVs) and cell free urine supernatant samples from healthy donors, patients with benign and malignant prostate tumours was done using miRCURY LNA miRNA qPCR Panels (Exiqon, Denmark). Sets of miRNAs differentially expressed between the donor groups were found in urine EVs and urine supernatant. Diagnostically significant miRNAs were selected and algorithm of data analysis, based on expression data on 24-miRNA in urine and obtained using 17 analytical systems, was designed. The developed algorithm of data analysis describes a series of steps necessary to define cut-off values and sequentially analyze miRNA expression data according to the cut-offs to facilitate classification of subjects in case/control groups and allows to detect PCa patients with 97.5% accuracy.

## Introduction

Timely detection of neoplasms is the key to successful tumor therapy. Thus, the urgent task of modern diagnostic medicine is the detection of cancer at the early stages of development (pre-cancer and early cancer), monitoring of the effects of anticancer therapy and detection of metastases and recurrences [1, 2].

Current methods of instrumental analysis including X-ray scans and magnetic resonance tomography, endoscopy and ultrasonography are not sufficient to detect the early stages of the disease, produce an excessive amount of false positive results and have limited population coverage, due to low productivity, requirements for highly qualified staff and use of expensive equipment [2, 3, 4].

The use of tumor tissue as a source of cancer biomarkers is only possible if the tumor has already formed, has been detected and can be easily sampled. It can be critical for assignment of targeted therapies and optimization of therapy regimes, but not suitable for early diagnosis [4]. Besides, biopsy does not necessarily provide a comprehensive representation of tumor phenotype due to the clonal nature and heterogeneity of cancer. In this regard, the detection of cancer markers in such readily available biological fluids as peripheral blood or urine can be a very promising approach to cancer diagnosis. Nevertheless, this liquid biopsy is still insufficiently realized in modern clinic practice, while immunohistochemical analysis of traditional extracellular protein oncomarkers is still widely used despite their lackluster specificity and sensitivity. Poor performance of the latter is also attributed to the tumor heterogeneity and slow growth rate, since the detectable amount of the protein antigen can be secreted into blood or urine only if tumor reaches a considerable size. Currently, United States Preventive Services Task Force (USPSTF) does not recommend the prostate specific antigen (PSA) as a marker for PCa screening due to over diagnosis and the absence of the effect on patient survival. The results of the PLCO and EPSPC studies induced by this committee clearly demonstrate that the clinic requires a more effective screening test for PCa [5].

One class of prospective PCa biomarkers is miRNA [6]—short (18–24 nucleotides), non-coding RNA molecules that act as posttranscriptional regulators of gene expression. It is known that miRNAs are involved in virtually all biological processes: embryonic development, proliferation, differentiation, aging, immune and stress response, genomic imprinting, key metabolic processes [7]. Tumour-derived miRNA can be found in the extracellular space and in biological fluids, which allows to use it to diagnose cancer and determine the tumour phenotype in a liquid biopsy format [8]. Extracellular miRNA remain stable due to packaging in nucleoprotein complexes or extracellular vesicles, such as microvesicles, exosomes or exosome-like particles [9, 10]. Mechanisms behind the miRNA loading and selective sorting into EVs are still largely unclear, but there are evidences that the analysis of miRNA content of EVs [2, 11, 12, 13] and nucleoprotein complexes [13, 14] present in the urine of PCa patients can be a promising tool in the development of cancer diagnostics. However, recent studies have shown that the expression of a single miRNA or several miRNAs is not sufficient to reliably diagnose cancer [15, 16]. Published data suggests that a comprehensive analysis of a panel comprised of several miRNA markers is required to achieve the desired efficiency of diagnostic tests [17, 18, 19]. The present study describes the search miRNA-based PCa markers in urine and the design of a diagnostic system based on the discovered markers.

## Materials and methods

Urine samples were obtained from 10 healthy men (HD, age range 48–65 years, mean age– 57.4 years), 10 patients with benign prostatic hyperplasia (BPH, age range 52–80 years, mean age– 57.4 years) and 10 previously untreated PCa patients (PCa, age range 56–82 years, mean age– 70.7). Diagnosis was confirmed based on clinical, morphological, radiological examination and the results of surgical intervention. Criteria for the PCa group were stage T_2-3_N_0_M_0_, PSA blood levels above 10 ng/ml and the absence of previous oncological diseases of the prostate or other localizations in the anamnesis. Criteria for the BPH group were clinical confirmation of the diagnosis and the absence of oncological diseases of the prostate or other localizations in the anamnesis. Men with a normal blood PSA level (below 2.8 ng/ml), no clinical history of oncological diseases and complaints from the urogenital system were included in the HD group. Samples were obtained from *“E*. *Meshalkin National medical research center” of the Ministry of Health of the Russian Federation*, (Novosibirsk, Russia). The work was conducted in compliance with the principles of voluntariness and confidentiality in accordance with the “Fundamentals of Legislation on Health Care”. The study was approved by the ethics committees of ICBFM SB RAS, *“E*. *Meshalkin National medical research center” of the Ministry of Health of the Russian Federation* (Novosibirsk, Russia) and *Novosibirsk Regional Oncology Center* (Novosibirsk, Russia) and written informed consent was provided by all participants.

### Urine fractionation and isolation of extracellular vesicles

Urine samples were stored at room temperature for no more than 3 hours before processing. Cell-free urine (supernatant after centrifugation 17000 g) and EVs fractions were obtained and characterized as described earlier [11].

### miRNA isolation

The modified acid phenol–chloroform extraction was used for miRNA isolation from 2 ml of urine supernatant [11].

miRNA extraction from 200 μl of EVs fraction (isolated from 20–30 ml of urine) was performed as described previously [20]. Isolated miRNA was precipitated by isopropanol, diluted in RNAse-free water and stored at -80°С.

### miRNA expression analysis

The comparative analysis of miRNA expression in urine fractions was conducted with a custom miRCURY LNA miRNA qPCR panel (Exiqon, Danmark) comprised of 67 miRNA from a pre-formed Urine Exosomes Focus Panel (Exiqon, Danmark) and additional cancer specific miRNAs, selected based on a literature analysis of miRNA expression in the prostate tissues and in urine of PCa patients (Table 1). A total of 84 miRNAs were included in the analysis.

**Table 1:** miRNA expression panel.

hsa-let-7a-5p	hsa-miR-126-3p	hsa-miR-191-5p	hsa-miR-24-3p	hsa-miR-346
hsa-let-7b-5p	hsa-miR-1285-3p	hsa-miR-193b-3p	hsa-miR-25-3p	hsa-miR-34a-5p
hsa-let-7c-5p	hsa-miR-130a-3p	hsa-miR-195-5p	hsa-miR-26b-5p	hsa-miR-375
hsa-let-7d-3p	hsa-miR-133a-3p	hsa-miR-19b-3p	hsa-miR-27b-3p	hsa-miR-378a-3p
hsa-let-7d-5p	hsa-miR-141-3p	hsa-miR-200a-3p	hsa-miR-29a-3p	hsa-miR-423-5p
hsa-let-7e-5p	hsa-miR-143-3p	hsa-miR-200b-3p	hsa-miR-29b-3p	hsa-miR-425-5p
hsa-let-7f-5p	hsa-miR-145-5p	hsa-miR-200c-3p	hsa-miR-29c-3p	hsa-miR-429
hsa-let-7g-5p	hsa-miR-146a-5p	hsa-miR-205-5p	hsa-miR-30a-5p	hsa-miR-451a
hsa-let-7i-5p	hsa-miR-148a-3p	hsa-miR-20a-5p	hsa-miR-30b-5p	hsa-miR-483-5p
hsa-miR-100-5p	hsa-miR-149-5p	hsa-miR-210-3p	hsa-miR-30c-5p	hsa-miR-484
hsa-miR-101-3p	hsa-miR-151a-5p	hsa-miR-214-3p	hsa-miR-30d-5p	hsa-miR-574-3p
hsa-miR-103a-3p	hsa-miR-15a-5p	hsa-miR-21-5p	hsa-miR-30e-3p	hsa-miR-582-5p
hsa-miR-106a-5p	hsa-miR-15b-5p	hsa-miR-221-3p	hsa-miR-30e-5p	hsa-miR-660-5p
hsa-miR-106b-5p	hsa-miR-16-5p	hsa-miR-222-3p	hsa-miR-31-3p	hsa-miR-92a-3p
hsa-miR-107	hsa-miR-17-5p	hsa-miR-22-3p	hsa-miR-31-5p	hsa-miR-93-5p
hsa-miR-10b-5p	hsa-miR-183-5p	hsa-miR-22-5p	hsa-miR-331-3p	hsa-miR-99b-5p
hsa-miR-125b-5p	hsa-miR-187-3p	hsa-miR-23b-3p	hsa-miR-33a-5p	сel-miR-39-3p

### Statistical analysis

Statistical analysis was performed using R language (version 3.3.1), Rstudio (version 1.0.136). Stats (v.3.3.1) was used to compare different groups. Ratio based normalization was applied to miRNAs and threshold cycle difference values (dCt) were calculated for each pair [21, 22]. To determine the difference between the median dCt values of different groups, the difference values of dCt (ddCt) values were calculated.

Boxes in box-and-whisker plots show mean and 1^st^ and 3^rd^ quartiles, whiskers indicate value range.

## Results

To study the distribution and representation of PCa-specific miRNAs in urine, a comparative analysis of 84 miRNA expression was performed in paired samples of EVs and cell-free urine supernatant from healthy men, patients with BPH and PCa using miRCURY LNA miRNA qPCR Panels (Exiqon Ltd, Denmark). On average, at least 70 of the 84 miRNAs were detected in each sample, with 72 miRNAs expressed in more than 70% of the samples. For the great majority of miRNAs the difference in expression between EVs and urine supernatant was quantitative, but a few miRNAs were predominantly present in only one of the fractions. The most obvious examples are miR-143-3p and miR-451a, which were present in cell-free urine supernatant and absent almost in all EVs samples. This can be due to the secretion or efflux of miR-451a as a part of protein and/or lipoprotein complexes from blood cells where it is highly expressed, followed by infiltration into the urine [23, 24]. However, during ultracentrifugation–based isolation of EVs the concentration of circulating miRNA complexes in the samples is reduced by more than twenty times.

At the first step, ratio normalization was applied to miRNA expression and dCt values were calculated in each pair of miRNAs [21, 22]. miRNAs detected in less than 80% of the samples (expression not detected or below the reliable detection threshold) were not included in the study. Remaining miRNAs were combined to produce approximately 3000 pairs. For each miRNA pair diagnostic sensitivity was determined at 100% specificity.

**Fig 1:**
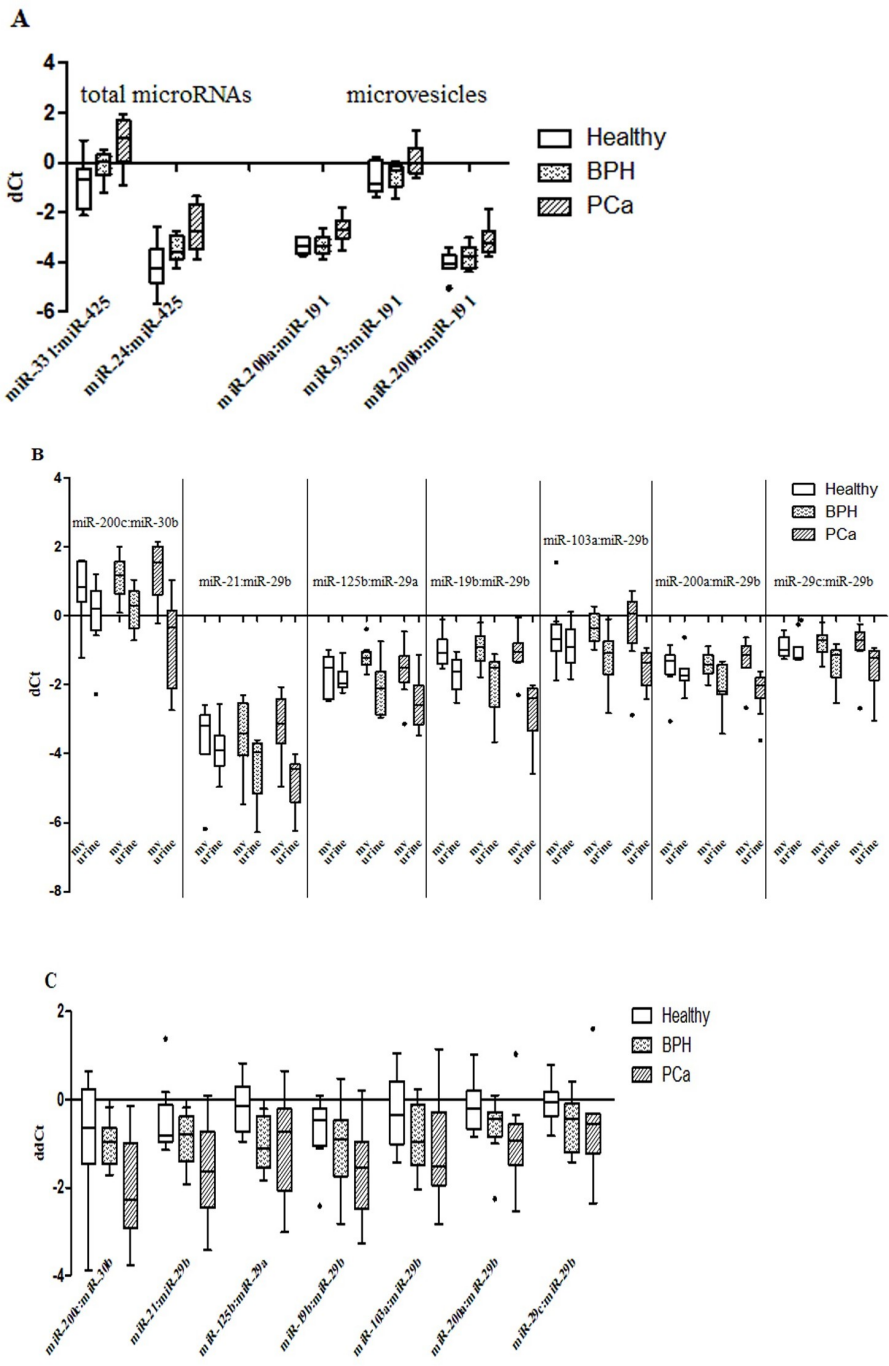
miRNA expression in the urine fractions of healthy men and patients with prostate diseases. Threshold cycle difference (dCt) values in miRNA pairs are shown. (A) dCt in EVs and total urine miRNA; (B) dCt for each urine miRNA fraction (mv–EVs; urine–supernatant after 17000g); (C) The relative expression of miRNA in cell-free fraction and in EVs. For between-groups comparisons threshold cycle difference (dCt) values in miRNA pairs are shown. For between-fractions comparisons dCt difference (ddCt) between supernatant and EVs fraction is shown.

Differences in miRNA expression between patient groups were found in both EVs and urine supernatant (Fig 1). Additionally, miRNA distribution was different between EVs and cell-free supernatant fractions (Fig 1).

Since individual miRNA were not enough to distinguish all three groups of patients with high sensitivity and specificity, we attempted to construct a classifier based on combinations of diagnostically significant miRNA pairs in order to improve the discrimination of PCa patients. To select miRNA pairs for the diagnostic system, we proposed a four-block data analysis algorithm.

**Fig 2:**
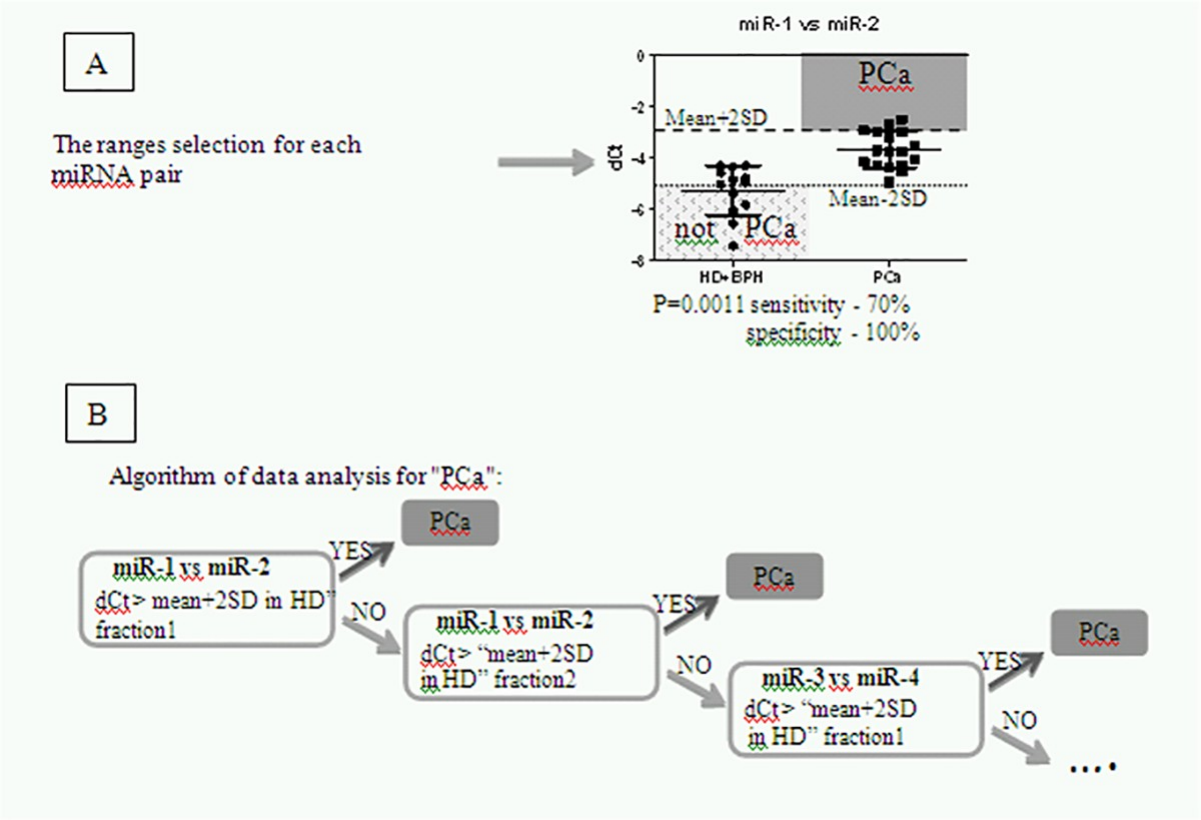
Scheme of data analysis for assured detection of PCa patients. (A) Range definition for a miRNA pair; (B) The decision tree of data analysis.

As opposed to a “computational algorithm” which is implemented in a form of a computer program or a standalone piece of software, here we use the term “algorithm” in a more broad and traditional sense. The algorithm presented here describes a series of steps necessary to define cut-off values and sequentially analyze miRNA expression data according to the cut-offs to facilitate classification of subjects in case/control groups. The miRNA pairs were assessed in order of highest sensitivity at 100% specificity also taking into account the level of markers expression. The design of diagnostic system includes the following steps:

Step 1. The data is divided in case (here, PCa) and control (here, HD+BPH) groups. If expression values are distributed normally, calculate the mean and standard deviation (SD) for the control group; if normality is rejected, calculate the median, 5% and 95% quantiles (Q5, Q95) instead. In the present study normal distribution was confirmed, thus mean and SD were calculated.
Step 2. Analyze the data on the miRNA expression in PCa patients samples. The cut-off value is determined as mean + 2SD or mean-2SD, obtained from the miRNA expression analysis in the "HD + BPH" group. If in the majority of values in patients were less than in healthy donors, «-2SD» is used, otherwise "+2SD" is used (Fig 2). Patients with dCt/ddCt values of a certain miRNA pair in the same biofluid fraction outside of the cut off are considered to have PCa.
Step 3. For the rest of the patients the expression of either the same miRNA pair in the other urine fraction or the next miRNA pair in the same / another urine fraction is analyzed. As previously, the cancer patient is identified by the comparison of dCt/ddCt with mean±2SD cut-off values obtained from the "HD + BPH" control group. Namely, the donor is considered to have PCa if dCt/ddCt values are above mean+2SD or below mean-2SD values.
Step 4. Steps 2 and 3 are repeatedly applied using the next miRNA pairs if the patient sample does not fall in the group of "PCa patients" on the previous steps. The algorithm is iterated until all patients were correctly classified or the pool of analytical systems is depleted.

An example "PCa” algorithm for detection of PCa patients is illustrated in Fig 2. The structure of the algorithm allows to use as little as possible number of analytical systems.

**Fig 3:**
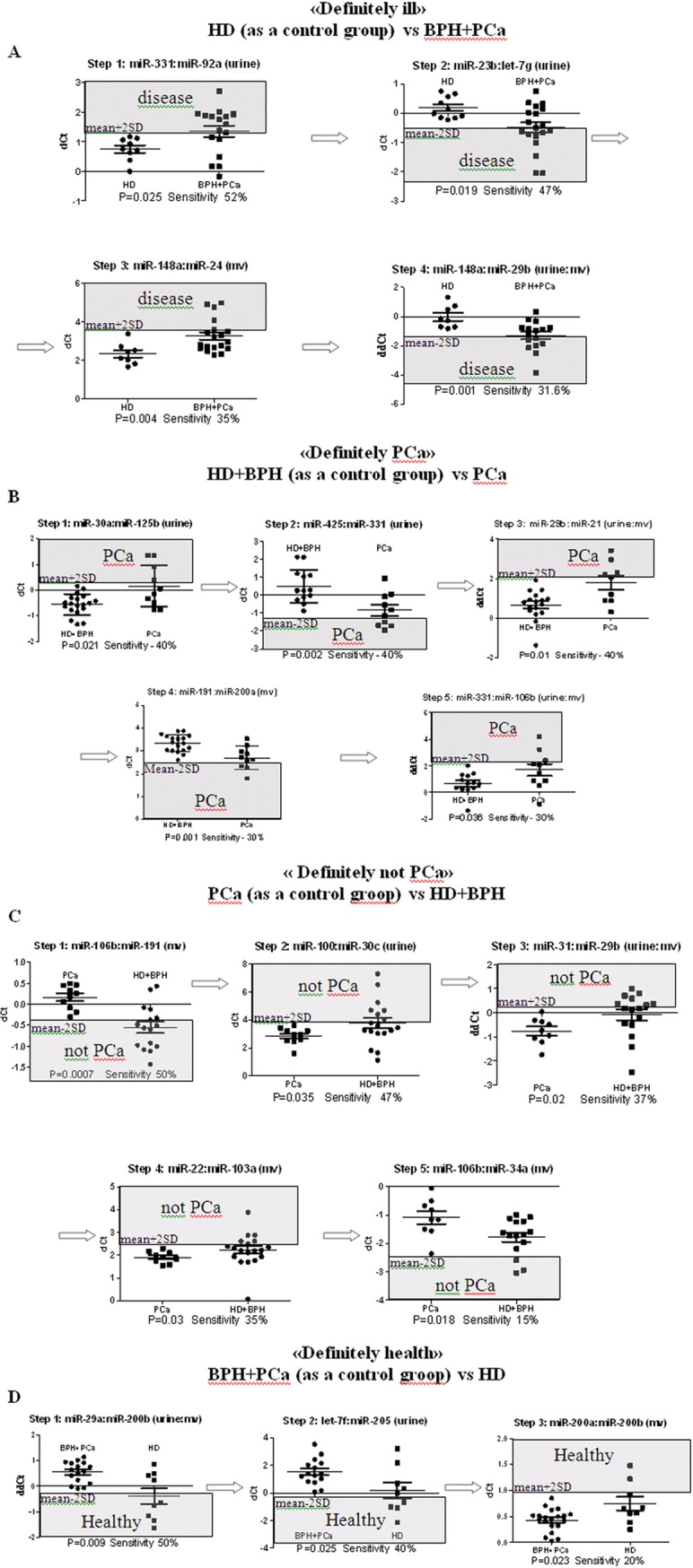
The developed data analysis blocks. (A) “Prostate disease” (HD (as a control group) vs BPH+PCa); (B) “PCa” (HD+BPH (as a control group) vs PCa); (C) «Not PCa» (PCa (as a control group) vs HD+BPH); (D) “Healthy” (BPH+PCa (as a control groop) vs HD).

To increase the reliability of the analysis, same approach can be repeated using different assembles of case-control groups. For, example alongside the described "PCa" algorithm, three blocks providing other diagnoses «Prostate Disease» (HD (control) vs BPH+PCa), «Not PCa» (PCa (control) vs HD+BPH), «Healthy» (BPH+PCa (control) vs HD), (Fig 3) were made. Since the distribution of joint control groups (e.g. HD+BPH) cannot be assumed a priori, it can be either treated as non-normal distribution or each of the subgroups can be analyzed separately and an overlap of the two ranges can be used for the purposes of the algorithm.

The proposed algorithm of diagnosis allowed us to perform the following: 1) determine patients with the diagnostic status «Prostate Disease» with absolute sensitivity and specificity (Fig 3; Table 2) using 4 analytical steps based on the expression of 7 miRNA in urine; 2) discriminate PCa patients from healthy donors and BPH patients with 90% sensitivity and 100% specificity (Fig 3; Table 3) using 5 analytical steps based on the expression of 9 miRNA in urine; 3) identify patients with the diagnostic status «Not PCa» with absolute sensitivity and specificity (Fig 3; Table 4) using 5 analytical steps based on the expression of 9 miRNA in urine; 4) determine patients with the diagnostic status «Healthy» with absolute sensitivity and specificity (Fig 3; Table 5) using 3 analytical steps based on the expression of 5 miRNA in urine;

**Table 2:** The example of using the diagnostic system for identification of men with prostate disease.

Sample	Step 1 miR-331: miR-92a (urine)	Step 2 miR-23b: let-7g (urine)	Step 3 miR-148a: miR-24 (mv)	Step 4 miR-148a: miR-29b (urine:mv)	No. of positive hits
Patients with BPH					
1	**V**				1
2	**V**				1
3	**V**	**V**	**V**		3
4			**V**		1
5			**V**		1
6			**V**		1
7	**V**	**V**		**V**	3
8				**V**	1
9		**V**			1
10	**V**				1
Patients with PCa					
1	**V**				1
2		**V**			1
3			**V**	**V**	2
4	**V**				1
5		**V**	**V**	**V**	3
6	**V**	**V**	**V**	**V**	4
7	**V**				1
8	**V**	**V**			2
9		**V**			1
10		**V**		**V**	2

HD (as a control group) vs BPH+PCa

**Table 3:** The example of using the diagnostic system for PCa diagnosis.

Patients with PCa	Step 1 miR-30a: miR-125b (urine)	Step 2 miR-425: miR-331 (urine)	Step 3 miR-29b: miR-21 (urine:mv)	Step 4 miR-191: miR-200a (mv)	Step 5 miR-331: miR-106b (urine:mv)	No. of positive hits
1	**V**					1
2						0
3	**V**	**V**	**V**			3
4				**V**	**V**	1
5		**V**			**V**	1
6	**V**	**V**		**V**		4
7						1
8	**V**	**V**	**V**	**V**	**V**	5
9			**V**			1
10			**V**			1

HD+BPH (as a control group) vs PCa

**Table 4:** The example of using the diagnostic system for identification of men without PCa.

Sample	Step 1 miR-106b: miR-191 (mv)	Step 2 miR-100: miR-30c (urine)	Step 3 miR-31: miR-29b (urine:mv)	Step 4 miR-22: miR-103a (mv)	Step 5 miR-106: miR-34a (mv)	No. of positive hits
Healthy donors						
1	**V**	**V**				2
2			**V**	**V**		1
3						1
4				**V**		1
5				**V**		1
6	**V**					1
7		**V**				1
8	**V**	**V**	**V**	**V**		4
9		**V**				1
10	**V**					1
Patients with BPH						
1	**V**		**V**			2
2	**V**	**V**	**V**			3
3	**V**	**V**	**V**	**V**		4
4	**V**					1
5		**V**				1
6	**V**					1
7					**V**	1
8	**V**				**V**	2
9			**V**	**V**		2
10					**V**	1

PCa (as a control groop) vs HD+BPH

**Table 5:** The example of using the diagnostic system for identification of healthy men.

HD	Step 1 miR-29a: miR-200b (urine:mv)	Step 2 let-7f: miR-205 (urine)	Step 3 miR-200a: mir-200b (mv)	The number of systems which showed a positive result
1	V			1
2			V	1
3	V		V	2
4	V			1
5	V			1
6		V		1
7		V		1
8		V		1
9		V		1
10	V			1

BPH+PCa (as a control groop) vs HD

Unfortunately, one PCa patient could not be accurately identified as “PCa” (Table 3), but was reliably classified into the “Prostate Disease” group (Table 2). Thus, overall accuracy of the developed data analysis algorithm was 97.5%.

The selected diagnostic systems displayed a certain degree of redundancy i.e. some patients can be classified as "PCa" (Table 3) or "Not PCa" (Table 4) based on the expression of one, two or more miRNA pairs. A diagnostic system based on the analysis of 3–5 miRNA pairs is more stable, and can reliably classify at least 90% of the patients. Moreover, the developed diagnostic systems yielded no false negative false positive results.

It is likely that with increased patient and control samples the high and low cut-off values could be refined to increase the effectiveness and sensitivity of the diagnostic system by allowing additional values both higher and below the values of mean ± 2SD of the control group into the diagnosis.

## Discussion

Prostate cancer is the second most diagnosed cancer among males worldwide [25]. The highest incidence rates are in the U.S. where it kills over 27000 men annually [26]. According to GLOBOCAN 2012 mortality rate in less developed countries is even higher. Despite a slight decrease in mortality from PCa since 1992, due to the widespread PSA testing of men's blood, the incidence of PCa continues to increase every year [27]. An overwhelming majority (99%) of all prostate cancers occur in men over 50 and are mostly slow-growing. In contrast, in younger men the development of aggressive PCa disease resulting in patient’s death is significantly more likely [27].

Unfortunately, in most cases early stage PCa is asymptomatic. The detection and local staging of PCa relies on digital rectal examination, detection of prostate specific antigen (PSA) in blood, transrectal ultrasonography, sextant biopsy, computed axial tomography scan, external and endorectal magnetic resonance tomography. The process of diagnostic decision making is quite complicated and insufficient sensitivity of these methods leads to underestimation of the stage in almost half of PCa patients [28, 29]. Thus, one of the major problems in the management of PCa is the development of diagnostic methods that can allow not only to detect the already formed prostate tumors, but also identify and monitor obligatory and facultative precancerous conditions and early stages of malignant tumor development.

One solution is offered by molecular genetics. In particular, the analysis of extracellular miRNA expression in body fluids has been considered a promising approach in the recent years. Currently, it is known that all forms of prostate neoplasms, including BPH have different miRNA expression profiles [DIANA-mirPath v.3.0]. Circulating miRNAs can be found in bodily fluids transported by EVs (including exosomes), high density lipoproteins and other ribonucleoprotein complexes [30, 31]. However, there is no general consensus on which particular miRNA fraction is better for the development of cancer diagnostics [32, 33]. In the most likely scenario, the use of certain fraction will be dictated by the properties of particular miRNA marker, since they are packaged in a somewhat sequence-dependent manner [11, 16, 32]. Several studies reported that analysis of circulating miRNAs allows to discriminate PCa patients and healthy donors with high sensitivity and specificity [11, 16, 34], but the performance was decreased when additional control groups, such as patients with BPH, were introduced into the experiment [35, 36]. It is likely to be explained by the physiological characteristics of men (age and general health similarities between PCa and BPH patients, organ-specificity rather than cancer-specificity of miRNA markers) and the course of PCa development. Namely, more than 50% of healthy (undiagnosed with prostate disorders) men over 50 years have BPH foci in the prostate, which are often microscopic, and well below the detection limit of first-stage diagnostics [37,38]. Additionally, PCa and BPH are to co-exist in the same patient, stressing the need for high biomarker specificity [38, 39, 40].

In the present work, we found differences in miRNA expression between donor groups in each of the urine fractions (Fig 1), as well as alterations in the miRNA distribution between EVs and urine supernatant, which correlated with prostate health (Fig 1). The incorporation of data obtained by the analysis of different urine fractions increases the diagnostic sensitivity of the system, which is especially important at the early stages of cancer when the tumor can’t be detected by instrumental and immunochemical methods.

Most significant differences in the miRNA expression between urine fractions were observed in PCa patients (38 miRNAs), whereas only 15 miRNA were differently expressed in the healthy donor group (15 miRNAs). Redistribution of miRNAs between the EVs and cell-free miRNA fractions occurred also with the development of BPH, exemplified by the distribution of miR-103a:miR-29b, miR-19b:miR-29b and miR-125b:miR-29b pairs (Fig 1). Two miRNA pairs miR-200a:miR-29b and miR-200c:miR-30b were expressed differently in EVs and urine supernatant only in PCa patients (Fig 1). Earlier Foj L. et al [16] revealed the alterations in the miRNA distribution between the cellular and exosomal urine fractions during the PCa development. For example, let-7c expression differed between healthy donors and PCa patients in the exosome fraction, but not in the urine cell sediment, while the expression of miR-141 and miR-214 was different in the urine sediment, but not in exosomes. The changes in the distribution of circulating miRNAs (let-7a, miR-141, mir-30c) between cell-free and exosomes fractions during PCa development in comparison with BPH were also observed in blood plasma [41]. All this may indicate that the packaging of at least some extracellular miRNAs is affected by the state of the prostate.

An analysis of the changes in miRNA expression within each of the urine fractions (supernatant or EVs) during PCa development (Fig 1) revealed significant differences for many miRNA pairs, for example, miR-200a: miR-191 between “PCa” and “Not PCa” (Fig 1). However, none of the miRNA pairs allowed to discriminate between all three donor groups with high specificity and sensitivity. The diagnostic potential of most of the differently expressed miRNAs was characterized earlier [11, 42, 43, 44, 45], but neither study has succeeded in achieving the highest sensitivity and specificity in distinguishing BPH and PCa patients, and healthy donors.

To improve the group discrimination, we have developed a diagnostic system based on the differentially expressed miRNAs (Fig 2) that allowed for classification of donors with a total accuracy of 97.5%. The system is based on the simplification of the data by employing arbitrary cut-off range accounting for the heterogeneity of the miRNA expression in the control group, so that only extreme changes are detected, ensuring high (close to absolute) specificity. The resulting loss in sensitivity is amended by using an array of markers (miRNA pairs) which cover the heterogeneity of group population. The diagnostic system can be further refined through analysis of larger donor groups, and additional miRNA markers can be introduced to increase diagnostic sensitivity and coverage, reliability of classification, and potentially allow for tumor phenotyping and therapy optimization.

This diagnostic panel includes 24 miRNAs (miR-21, -22, -23b, -24, -29a, -29b, -30a, -30c, -31, -34a, -92a, -100, -103a, -106b, -125b, -148a, -191, -200a, 200b, -205, -331, -425, let-7f, let-7g). Several of these miRNAs were previously implicated in PCa development, and diagnostic [13, 46] and prognostic [13, 47, 48, 49] properties of miR- -191, -425 and others have previously been reported [48]. Using available databases (DIANA, STRING, PANTHER) we conducted a bioinformatics analysis of 24 miRNAs used in all 4 blocks of the diagnostic system. According to DIANA these miRNAs are controlling 78 genes involved in PCa development. These genes are related to such molecular processes as binding to cellular components (41.7%, with 8.8% of them are related to *chromatin binding*, 28.1%—to *nucleic acid binding*, 63.2%—to *protein binding*), *catalytic activity* (38%, with 80.5% of them responsible for *transferase activity*), *receptor activity* (6.5%) and *signal transducer activity* (13.9%) according to PANTHER. In regards to biological processes, miRNA targets were associated with *cellular process* (23.3%, with 65.3% of them responsible for *cell communication*), *metabolic process* (21.8%), *response to stimulus* (15.6%), *biological regulation* (12.6%), and *developmental process* (7.3%, with 34.8% of them responsible for *cell differentiation*, 17.4%—for *cell death* and 17.4%—for *ectoderm development*).

**Fig 4:**
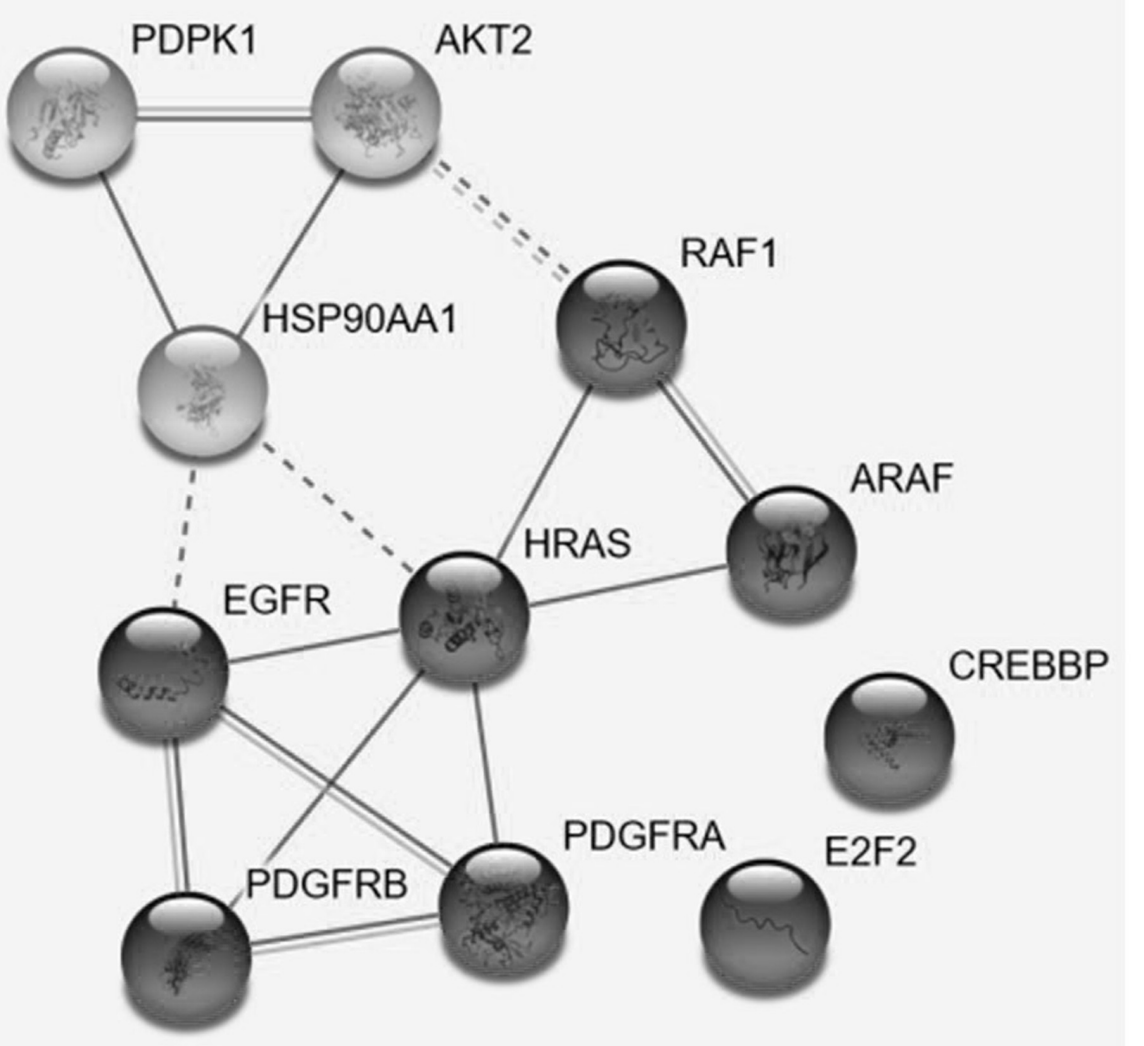
STRING data: interactional network of 10 genes, detected simultaneously in 3 "diseased" blocks («Prostate Disease», «PCa», «Not PCa») and missing in the block "Healthy" and one gene (HRAS), found only in the "Healthy" block.

From the 77 out of 78 genes, found in the STRING database, 73 were shown to participate in PCa development. Detailed analysis was performed for 10 genes which were shared targets of miRNAs in the three "diseased" blocks («Prostate Disease», «PCa», «Not PCa») but absent from the «Healthy» block, and 1 gene (HRAS) targeted exclusively by miRNAs the «Healthy» block. According to STRING, these 11 genes (Table 6) are responsible for PCa development and can be split into two groups: (Fig 4). Moreover, expression of these genes may be up- or downreguated depending on the type of cell line: PDGFRA (up), E2F2 (up+down), HSP90AA1 (up), RAF1 (up), EGFR (up), ARAF (no information), AKT2 (up), PDPK1 (up+down), CREBBP (up), PDGFRB (up+down), HRAS(up) according to published data. According to PANTHER PDGFRA, PDGFRB, E2F2, EGFR, CREBBP and HRAS are responsible for binding; ARAF, RAF1, HRAS, AKT2, CREBBP and PDPK1 are responsible for *catalytic activity*; PDGFRB, PDGFRA and EGFR are related to both *receptor activity* and *signal transducer activity*. In regards to the cellular component, the genes were associated with *cell part*, *macromolecular complex* and *membrane* in equal proportions (33.33% each).

**Table 6:** Functions of genes, detected simultaneously in 3 "diseased" blocks («Prostate Disease», «PCa», «Not PCa») and missing in the "Healthy" block and one gene (HRAS), found only in the block "Healthy" (STRING data).

RAF1	*V-raf-1 murine leukemia viral oncogene homolog 1; Serine/threonine-protein kinase that acts as a regulatory link between the membrane-associated Ras GTPases and the MAPK/ERK cascade*, *and this critical regulatory link functions as a switch determining cell fate decisions including proliferation*, *differentiation*, *apoptosis*, *survival and oncogenic transformation*. *RAF1 activation initiates a mitogen-activated protein kinase (MAPK) cascade that comprises a sequential phosphorylation of the dual-specific MAPK kinases (MAP2K1/MEK1 and MAP2K2/MEK2) and the extracellular signal-regulated kinase*.
PDGFRA	*Platelet-derived growth factor receptor*, *alpha polypeptide; Tyrosine-protein kinase that acts as a cell-surface receptor for PDGFA*, *PDGFB and PDGFC and plays an essential role in the regulation of embryonic development*, *cell proliferation*, *survival and chemotaxis*. *Depending on the context*, *promotes or inhibits cell proliferation and cell migration*. *Plays an important role in the differentiation of bone marrow-derived mesenchymal stem cells*. *Required for normal skeleton development and cephalic closure during embryonic development*.
PDGFRB	*Platelet-derived growth factor receptor*, *beta polypeptide; Tyrosine-protein kinase that acts as cell-surface receptor for homodimeric PDGFB and PDGFD and for heterodimers formed by PDGFA and PDGFB*, *and plays an essential role in the regulation of embryonic development*, *cell proliferation*, *survival*, *differentiation*, *chemotaxis and migration*. *Plays an essential role in blood vessel development by promoting proliferation*, *migration and recruitment of pericytes and smooth muscle cells to endothelial cells*. *Plays a role in the migration of vascular smooth muscle cells*.
CREBBP	*CREB binding protein; Acetylates histones*, *giving a specific tag for transcriptional activation*. *Also acetylates non-histone proteins*, *like NCOA3 and FOXO1*. *Binds specifically to phosphorylated CREB and enhances its transcriptional activity toward cAMP-responsive genes*. *Acts as a coactivator of ALX1*. *Acts as a circadian transcriptional coactivator which enhances the activity of the circadian transcriptional activators- NPAS2-ARNTL/BMAL1 and CLOCK- ARNTL/BMAL1 heterodimers*. *Acetylates PCNA; acetylation promotes removal of chromatin-bound PCNA*.
EGFR	*Epidermal growth factor receptor; Receptor tyrosine kinase binding ligands of the EGF family and activating several signaling cascades to convert extracellular cues into appropriate cellular responses*. *Known ligands include EGF*, *TGFA/TGF-alpha*, *amphiregulin*, *epigen/EPGN*, *BTC/betacellulin*, *epiregulin/EREG and HBEGF/heparin-binding EGF*. *Ligand binding triggers receptor homo- and/or heterodimerization and autophosphorylation on key cytoplasmic residues*. *The phosphorylated receptor recruits adapter proteins like GRB2 which in turn activates complex downstream signaling cascades*.
HRAS	*v-Ha-ras Harvey rat sarcoma viral oncogene homolog; Ras proteins bind GDP/GTP and possess intrinsic GTPase activity*
HSP90AA1	*Heat shock protein 90kDa alpha (cytosolic)*, *class A member 1; Molecular chaperone that promotes the maturation*, *structural maintenance and proper regulation of specific target proteins involved for instance in cell cycle control and signal transduction*. *Undergoes a functional cycle that is linked to its ATPase activity*. *This cycle probably induces conformational changes in the client proteins*, *thereby causing their activation*. *Interacts dynamically with various co-chaperones that modulate its substrate recognition*, *ATPase cycle and chaperone function*.
PDPK1	*3-phosphoinositide dependent protein kinase-1; Serine/threonine kinase which acts as a master kinase*, *phosphorylating and activating a subgroup of the AGC family of protein kinases*. *Its targets include- protein kinase B (PKB/AKT1*, *PKB/AKT2*, *PKB/AKT3)*, *p70 ribosomal protein S6 kinase (RPS6KB1)*, *p90 ribosomal protein S6 kinase (RPS6KA1*, *RPS6KA2 and RPS6KA3)*, *cyclic AMP-dependent protein kinase (PRKACA)*, *protein kinase C (PRKCD and PRKCZ)*, *serum and glucocorticoid-inducible kinase (SGK1*, *SGK2 and SGK3)*, *p21-activated kinase-1 (PAK1)*, *protein kinase PKN (PKN1 and PKN2)*.
E2F2	*E2F transcription factor 2; Transcription activator that binds DNA cooperatively with DP proteins through the E2 recognition site*, *5’-TTTC[CG]CGC- 3’ found in the promoter region of a number of genes whose products are involved in cell cycle regulation or in DNA replication*. *The DRTF1/E2F complex functions in the control of cell-cycle progression from g1 to s phase*. *E2F2 binds specifically to RB1 in a cell-cycle dependent manner*.
ARAF	*V-raf murine sarcoma 3611 viral oncogene homolog; Involved in the transduction of mitogenic signals from the cell membrane to the nucleus*. *May also regulate the TOR signaling cascade*.
AKT2	*V-akt murine thymoma viral oncogene homolog 2; AKT2 is one of 3 closely related serine/threonine- protein kinases (AKT1*, *AKT2 and AKT3) called the AKT kinase*, *and which regulate many processes including metabolism*, *proliferation*, *cell survival*, *growth and angiogenesis*. *This is mediated through serine and/or threonine phosphorylation of a range of downstream substrates*. *Over 100 substrate candidates have been reported so far*, *but for most of them*, *no isoform specificity has been reported*. *AKT is responsible of the regulation of glucose uptake*.

Many of miRNAs (15 of 24) featured in the diagnostic system are involved in the regulation of the p53 signal pathway, a smaller number of miRNAs target other important pathways, associated with PCa, including TNF– 2 genes, AMPK– 2 genes, mTOR- 1 genes, MAPK -7 genes, ERBB– 7 genes (DIANA database). This shows that an analysis of a miRNA panel constructed of these miRNA can potentially reveal the status of multiple key regulatory pathways, increasing the value and relevance of the diagnostic system. Apart from their diagnostic application, some of identified miRNAs and their target genes are perspective targets for PCa treatment and indicators of long-term prognosis. For example, miR-331 and miR-125, included in the «PCa» block (miR-331 is also present in the «Prostate Disease» block), are the only miRNAs from the diagnostic system that regulate the ERBB2 gene, which encodes for the transmembrane protein from the epidermal growth factor receptor family (HER/EGFR/ERBB) (DIANA database). According to DIANA database several miRNAs from the «PCa» block, including miR-331 and miR-125, target genes, such as BRAF, GSK3B, E2F1, CDKN1A, CDKN1B, EGFR, AKT2, MAPK1, which interact with the ERBB pathway and are involved in the development of PCa. The ERBB family is among the most frequently dysregulated receptor tyrosine kinases in neoplasms, for example in breast, cervix, colon, endometrial, esophageal, lung, and pancreatic cancers [50, 51, 52]. Overexpression of ERBB2/HER2 promotes proliferation, metastasis, angiogenesis, tumour progression, suppresses apoptosis and correlates with poor survival of PCa patients (see [11] for review). This gene is a target of several PCa therapeutics. Celecoxib, a COX-2 inhibitor, has anti-androgen properties mediated by ErbB receptor kinase inhibition [53]. Authors suggest that as ErbB inhibitor, celecoxib can be used at the pre-cancerous stage to prevent disease progression, or combined with anti-androgen therapy in patients with hormone-sensitive disease [53]. Another agent which targets ERBB2 is scFv(FRP5)-ETA, based on PCa cell lines study it was suggested for treatment of tumours with high HER2 expression [54]. Androgen receptor (AR) and ErbB pathways are known to interact, partially overlap and converge on targets downstream of each other’s signaling cascades to promote the survival of androgen-independent cells, leading to androgen-independent PCa tumours [55, 56, 57]. Inhibition of interactions between the two pathways could convert androgen-independent PCa back to a hormone-sensitive state. Offering a prospective strategy of the PCa management [58]. In a mouse PCa xenograft model, androgen-independent xenografts expressed higher levels of HER-2 than their androgen-dependent counterparts [59]. Besides, Her-2/neu-positive patients with clinically localized PCa are at a higher risk for disease progression, metastasis and PCa-induced death. Furthermore, Her-2/neu expression can be used alongside clinicopathologic parameters to assess the long-term PCa prognosis [60]. In view of the above, the development of ErbB targeted chemopreventive and chemotherapeutic agents is very likely.

Thus, selected miRNAs are involved in regulation of key oncogenic pathways in PCa development, which can potentially extend the utility of the suggested diagnostic system beyond cancer detection and diagnosis. This is in accordance with the notion that miRNAs can be potent cancer biomarkers [6]. Several approaches for miRNA-based cancer biomarker selection have been published to date [7–10]. Many of the suggested panels have failed to achieve the high sensitivity and specificity; others are based on the analysis of changes occurring in the tumor tissue and can’t be translated to liquid biopsy. Moreover, as the above literature analysis shows, selected miRNAs can be potentially used not only as PCa markers, but as keys to find genes, deregulated during PCa progression, and to develop strategies of the PCa management.

## Conclusion

The expression and distribution of 84 miRNAs in two urine fractions (EVs and cell-free supernatant) was studied in healthy donors, patients with BPH and PCa. An approach to the development of a miRNA-based diagnostic system was proposed and resulting panels used to classify PCa and BPH patients and HD with 100% specificity and 97.5% accuracy. The diagnostic system based on the expression of 5 miRNA pairs (miR-30a: miR-125b; miR-425: miR-331; miR-29b: miR-21; miR-191: miR-200a; miR-331: miR-106b), can be potentially used to identify PCa and aid in therapy optimization.
